# Probe into the targeted poverty mitigation policy in China based on causal inference: Evidence from Chongqing in the Three Gorges Reservoir region

**DOI:** 10.1371/journal.pone.0244928

**Published:** 2021-01-05

**Authors:** Min Zhu, Chuanmin Shuai

**Affiliations:** School of Economics and Management, China University of Geosciences (Wuhan), Wuhan City, China; School of Economics, Xiamen University, China, CHINA

## Abstract

A quantitative analysis of the panel data of 21 districts in Chongqing City in the Three Gorges Reservoir region from 1998 to 2015 was conducted to determine the influence of China’s focused poverty reduction policy in terms of causal inference. Specifically, the effects of this policy at the initial phase of execution through break-point regression; the impacts of this policy during the implementation period by using an instrumental variable panel regression; and comparative studies of the policy impacts by using the generalized synthetic control method. The outcomes showed that: (1) China’s state policy for specific poverty mitigation has a substantial impact on reducing poverty, which has been observed from the start of the enactment, namely, peasants' net income per capita from 2011 to 2013 increased by 13.9%, as compared to that during 2008–2010. Meanwhile, the net earnings of each farmer grew by 22% from 2011 to 2015 throughout the implementation phase of the policy, as opposed to that in 1998–2010. (2) Economic behavior in the marketplace and agricultural output figure of impoverished regions has significantly improved farmers' individual net income. (3) The comparative study further proves that the poverty mitigation policy has the effect of reducing poverty. Based on these findings, it is proposed that China should increase investment to consolidate its poverty alleviation policy in the most poverty-stricken areas, activate rural markets and develop agricultural production at the local level to accelerate poverty reduction in the region. The method of causal inference was utilized as an innovative method to study China’s focused poverty reduction policy for both immediate and permanent effects. It is the first time to use the generalized integrated control method, which is a causal inference frontier method, to further verify the effectiveness of poverty reduction policies.

## Introduction

Anti-poverty have always been important issues of common concern to all mankind. The poverty problem worldwide is still severe. Hence, the 2030 Agenda for Sustainable Development issued by the United Nations lists "eliminating all forms of poverty in the world" as the top of the 17 sustainable development goals. China has unremittingly committed to poverty alleviation and contributed greatly to world poverty reduction. From 1990 to 2018, China's impoverished population has declined by 737 million, contributing over 70% of the global poverty reduction results, which has made the country leading with the highest poverty reduction among citizens globally [1]. Meanwhile, China’s rural development tasks are still arduous. China still has 16.6 million rural poor people, who are distributed in deeply impoverished areas. Therefore, the poverty mitigation in those regions is the current aim of poverty eradication challenge. To mitigate the issue, China implemented the targeted poverty alleviation policy in 2011. This policy targets counties that are deemed to be poverty-stricken and uses differential and effective methods to carry out targeted identification, assistance, management and assessment of rural poverty. There are several features of China’s targeted poverty alleviation policy. Firstly, it emphasizes precision, i.e., it makes poverty alleviation targets more accurate in ways such as investigating and analyzing the causes of poverty on a household basis in order to propose anti-poverty strategies for each and every impoverished household. Secondly, it focuses on narrowing regional development differences.

The vast majority of poor districts in China are mostly located in the country's central region, western mountainous regions, and southwestern and northeastern regions. By the end of 2019, the rural impoverished populace were 3.23 million in the western region, 1.81 million in the central region, and 0.47 million in the eastern region. Compared with those in 2018, the number of people in poverty is 5.93 million, 4.16 million and 1 million fewer for the regions in the west, central and the east, respectively. The total population of destitute in rural China had declined from 98.99 million to 5.51 million from 2012 to 2019, with a collective decrease of 93.48 million (China’s National Bureau of Statistics, 2020). Currently, the poorest areas in China are clustered in contiguous and difficult-to-live areas. One of these areas is the TGRR (Three Gorges Reservoir Region, the same as above) which is situated in the Qinba and Wuling Mountains. The TGRR in Chongqing covers 21 counties, including 9 poor and 12 non-poor districts. Within the poverty is particularly severe in nine poverty-stricken counties located in the hinterland of Qinba and Wuling Mountains under the administration of Chongqing Municipality of China. Their development is slow due to the inconvenience of transportation and the constraints of resources. Their self-sufficiency of agricultural production is prominent, their production mode is relatively backward, and their living standards are low. In comparison, the remaining 12 non-poor counties have better access to transportation with faster economic progress.

The implementation of anti-poor policy has led to significant increases in the efficacy of poverty mitigation [2]. From the perspective of the efficiency of poverty relief policies, Ali et al. [3] established an effective index system to explore the influence of Iran's financial and taxation programs on poverty. The results showed that Iran's policies on these areas can significantly reduce poverty. Li [4] believed that China's poverty reduction policy is effective. From the view of sustainability of poverty alleviation policies, related policies have substantially boosted the individual net earnings of households, and the effects of poverty alleviation have continued over time [5–7]. Market-based reforms have reduced rural poverty [8]. Market incentives and services can improve the lives of people in poverty-stricken areas. For most of the poorest areas, agriculture is an essential component of an effective development strategy [9]. Rapid agricultural growth promotes poverty mitigation and economic progression [10]. In the current study, the impact of focused poverty reduction policy was primarily based from a qualitative or farmers’ perspective [11–13]. Previous studies have focused on the TGRR locked in poverty, water contamination and agricultural development [14–17].

After analyzing the existing literature, the current researches have the following limitations: Firstly, the lack of an objective method to study the effectiveness of poverty reduction policies. At present, scholars adopt artificial index systems to study the success of this policy, which is subjective. Secondly, the lack of dynamic assessments for effects of the poverty relief policy. At present, most scholars study poverty reduction policy from the perspective of the effectiveness or long-term. Such research is relatively scattered, and lacks in the research that systematically takes time as a cut-in point and makes dynamic research on effects of the poverty reduction policy. Thirdly, the lack of comparative research to comprehensively measure the poverty reduction effects of policies in favor of the poor. Scholars study the influences of poverty reduction policies mainly from before and after the implementation of poverty reduction policies in poor counties. These studies lack the comparison between destitute and rich counties that have never implemented poverty reduction policies in order to comprehensively evaluate the poverty reduction effects of pro-poor policies.

The objectives of this study are as follows: Firstly, the breakpoint regression method was used to study the policy impacts in the preliminary phase of the enactment of poverty reduction policies. And then, the data-driven method was utilized to identify the effects of time in the early stage of the enforcement of the policies. Secondly, time was regarded as the research entry point to assess the overall influence of the poverty mitigation policy from the immediate and the permanent effect. Thirdly, the poverty reduction outcome of these policies was further verified based on a comparative study. Fourthly, the causal inference was used to study the influence of reducing poverty through the focused poverty reduction policy.

## Research design

### Data and variable selection

We used the panel data from 21 counties in the TGRR of Chongqing from 1998 to 2015 to analyze the success of the focused poverty mitigation policy with 9 poor counties (namely, Fengdu, Fengjie, Kaixian, Yunyang, Wanzhou, Wulong, Wushan, Wuxi, and Shizhu), and 12 non-poor counties (including Jiulongpo, Dadukou, Changshou, Banan, Beipei, Jiangbei, Jiangjin, Shapingba, Zhongxian, Nan'an District, Fuling, Yubei). Selected data are collated from the "China County (City) Social Economic Statistical Yearbook", "China Regional Economic Statistical Yearbook", "China Statistical Database of Economic Information Network" and statistical bulletin of districts and counties. This paper has assessed the immediate and lasting impacts of the enactment of the focused poverty reduction policy, which was first implemented in 2011. For this study, the dependent variable is the individual net pay of farmers, which is utilized to analyze the effect of this policy in the new era. The reason is that since 1990, the World Bank has used income as a standard to measure poverty. Since 1998, China has taken income as the measure of poverty. Since various researchers selected the personal net earnings of farmers to evaluate the impact of this pro-poor policy [18, 19].

Meanwhile, gross agricultural product and market profitable activity in poor areas are the independent variables, which embody the macroeconomic measures that influence the success of the poverty mitigation policy. In this study, the market economic activities among those independent variables are measured by the sum of merchandise sales of consumer products. Xiong et al. [20] and Jian et al. [21] used these as a proxy indicator of market economy activities. The total retail sales of consumer goods refers to the total amount of sales of agricultural means of production sold to rural areas by industrial departments and direct retails by farmers to urban residents. It reflects the development effect of the rural market economy [22]. Previous studies have proven that agricultural gross output in poverty-stricken areas include beneficial impacts on poverty reduction [23, 24]. From the above analysis, we can see that the poverty alleviation policy indirectly affects the increase of farmers’ income through the gross value of agricultural production and the active degree of market economy. Therefore, we used these two variables as the explanatory variables of the increase of farmers’ individual net earnings. The instrumental variable is road network density of poor counties used as the instrumental variable of the poverty alleviation policy, because it is learned from other scholars' approaches. For example, Card [25] used the distance from the interviewee's home to the nearest university as the instrumental variable of the education in order to analyze whether education can increase the income and status of individuals. In the study of institutional analysis [26], the distance from each country to the equator was used as the instrumental variable. Hence, we have used the road network density as basis for the poverty alleviation policy. The calculation for this variable is the road mileage divided by the area in accordance with Statistical Yearbook approaches, which is a measure of regional traffic convenience [27, 28]. Since the value of these variables (the individual net earnings of farmers; output value of agriculture and the overall retail sales of consumer products) are relatively large, the logarithmic transformations of these raw data were made for the convenience of calculation.

### Poverty reduction path of the focused poverty mitigation policy

**Fig 1:**
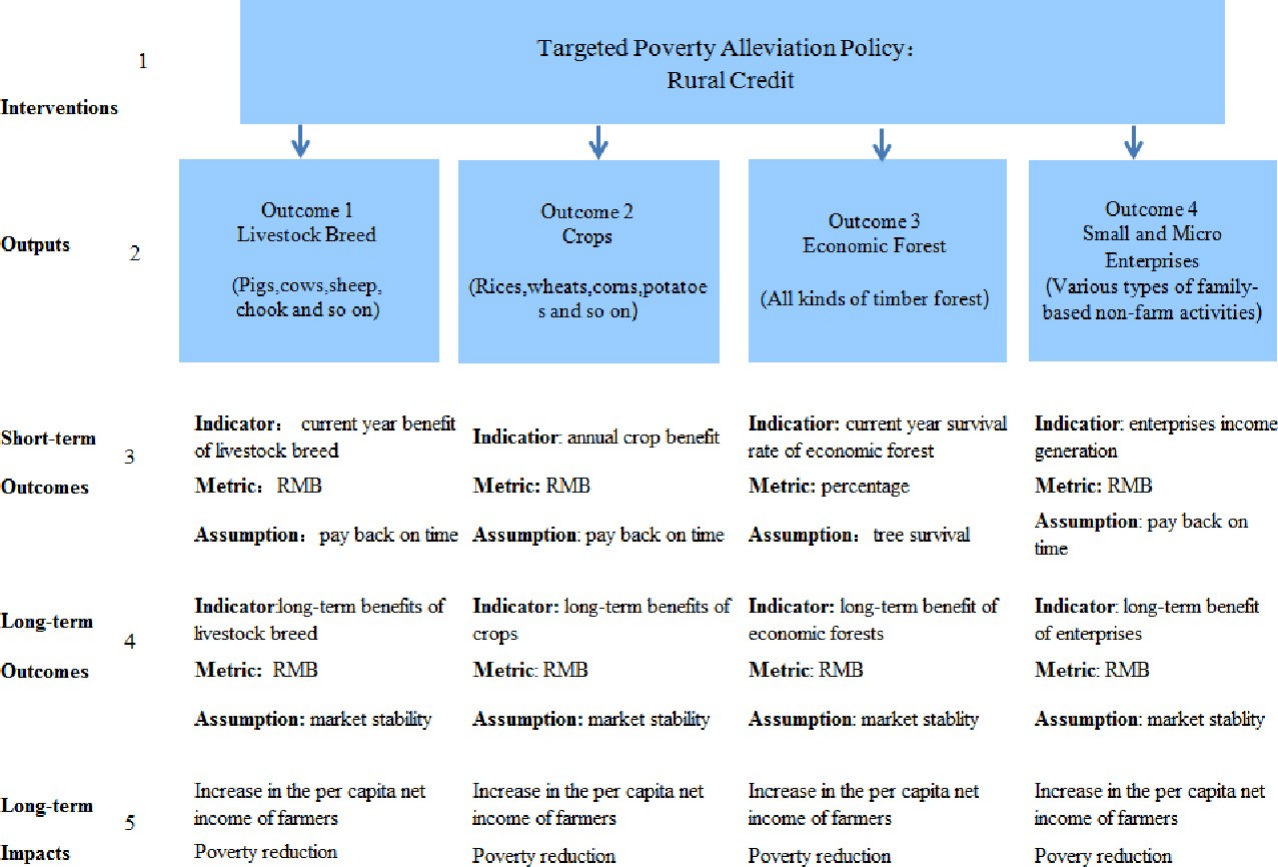
Poverty reduction path of the focused poverty mitigation policy.

The poverty reduction path of the focused poverty mitigation policy is explained by the theory of change (ToC). The theory of change offers a theoretical basis for the poverty reduction project of the World Bank. It describes the types of intervention (an anti-poverty project or a policy) that generates the results illustrated in the change map’s pathway. Every consequence in the pathway of change is linked to interference, exposing the commonly intricate web of activities that is necessary to cause the change [29]. Through a series of "input-output-result-impact" causal chains, the ToC elaborates the poverty reduction path of the specific poverty reduction policy. We took rural credit in this type of policy as a model to illustrate the poverty reduction path. For example: A poor household obtained a micro loan of 3,000 Yuan (poverty alleviation policy intervention) and bought five lambs (output). After one-year breeding, the poor household sold five sheep and earned 4,000 Yuan (result). The poor household repeated the pattern to earn a steady income. Finally, the poor household eradicates poverty (short-term impact). Through sheep breeding for profit, poor households achieve an annual increase in their per capita net income, and eventually rise out of poverty (long-term impact). The same applies to ToC for other pro-poor project activities policy. The poverty reduction path of the focused poverty mitigation policy is presented in Fig 1.

### Impact of the focused poverty mitigation policy at the initial phase of enforcement

The impacts of the focused the poverty reduction policy was analyzed. Systematic difference is not assessed in all the factors for impoverished districts in 2010 and 2011 before and during the policy implementation, respectively. Thus, the break-point regression could be utilized to determine the impacts of the policy at the initial phase of its implementation. The breakpoint for this article is 2011, when the targeted poverty reduction policy started.

The break point regression equation is shown below:

$$y_{i,t}=x_{i,t}^{'}\beta +\gamma_{i}t+\delta D_{i}+\mu_{i}+\varepsilon_{i,t}$$ (Eq 1)

$$D_{i}=\{\begin{array}{ll} 1 & \mathrm{if}\;x_{i}\geq 2011\\ 0 & \mathrm{if}\;x_{i}<2011 \end{array}$$

*where*, i signifies the national level poor districts (Wanzhou, Fengdu, Fengjie, Kaixian, Shizhu, Wulong, Wushan, Wuxi and Yunyang) in the TGRR of Chongqing. D_i_ is the processing state variable, and x_i_ is the driving variable expressed as a year. If the povery relief policy in 2011 is the threshold, then Di is assigned as 1 when x_i_ is no less than year 2011 meanwhile observed individuals enter treatment group; otherwise, D_i_ is assigned as 0. y_i,t_ indicates farmer net income per capita, δ measures the influence of D_i_ (poverty alleviation policy) on y_i,t_, namely the causal link between them. $x_{i,t}^{\prime }$ are covariates, namely: output value of agriculture and market economic profitable activity. This study presents the outcomes of the non-paramentric estimation, calculated by the quadratic and triangular kernel.

The empirical findings in Table 1 depicts that the poverty reduction effects of the pro-poor policy are significant; with the value are 0.139 and 0.138 by the that is by the triangular and quadratic kernel estimation, respectively. Therefore, the poverty reduction effect at the start of the enactment of the focused poverty mitigation policy is 0.139.

**Table 1:** Results of impacts of the focused poverty reduction policy at the early phase of enforcement.

	(1)lnrural (tri)	(2)lnrural (epa)
Conventional	0.139*	0.138*
	(0.055)	(0.055)
Bias-corrected	-0.059	-0.059
	(0.055)	(0.055)
Robust	-0.059	-0.059
	(0.094)	(0.094)

**Note:** (1) *, **, and *** indicate the results are statistically significant at the levels of 5%, 1% and 0.1%, respectively. (2) Conventional is a traditional estimate; Bias-corrected is an estimate of deviation correction; Robust is a robust estimate in case of When Robust considers the deviation A robust estimate. (3) tri is triangular kernel; epa is quadratic kernel.

In the following empirical analysis, we judge the time when the impacts appeared at the start of the enforcement of the poverty reduction policy. Cattaneo et al. [30] suggest that the time of the policy effect can be judged by the sudden change of the data distribution on both sides of the breakpoint. The optimal bandwidth can be calculated by the method of CV, IK and CCT. Calonico et al. [31] believe that the optimal bandwidth obtained by the CV and IK method is too large, resulting in a deviation of the corresponding confidence interval. However, the CCT method can correct the deviation caused by the excessive bandwidth. Therefore, We used CCT method to estimate the time when the policy effect appears, and its estimated value is the optimal bandwidth of breakpoint regression.

From the observed results in Table 2, it can be seen that the estimated value of the CCT method is 3.219. It means that the three-year sample near the breakpoint in 2011 is used to estimate the short-term effects of policy, that is, the short-term outcomes of the poverty mitigation policy occurred in the time interval from 2011 to 2013.

**Table 2:** Empirical results of the emergence time of the anti-poor policy’s effect.

Method	h	b	rho
CCT	3.291601	6.486192	0.5074783

In the S1 Appendix, we presented the results of the breakpoint regression’s robustness test. The results indicate that the increase in the farmers’ pure income is only caused by the enactment of the focused poverty reduction policy, rather than the covariates (profitable market activity and overall cost of agricultural production in poor regions). Therefore, it passed the said test.

### Permanent effect of China’s poverty mitigation policy

Breakpoint regression is only capable to calculate the processing impacts if close to the breakpoint, thus cannot be utilized for those far from the division. Specifically, it can only approximate the influence of the focused poverty mitigation policy within the year of implementation. Hence, it is incapable to determine the outcomes during the duration of the policy, that is, across the years 2011 to 2015. We have used the instrumental variables panel regression method to assess the policy effects throughout the targeted alleviation phase (2011–2015) in order to fulfill the causal inference of policy beyond the breakpoint. The impoverished areas in the TGRR have poor resource endowment due to their location in mountains with inconvenient transportation links and poor natural resources. In ordinary panel regressions, the factor of resource endowment is omitted, so we have solved the problem of missing variable and realized causal inference by the method of instrumental variables. Instrumental variable (IV) can identify causal inference regarded as a random experiment. Doing so, avoids having too many control variables in regressions, and also solves the problem of omitted or unknown control variables [32].

The instrumental variable panel regression formula is shown below:

$$y_{i,t}=x_{i,t}^{'}\beta +\gamma_{i}t+D\delta_{i,t}+\alpha Z_{t}+\mu_{i}+\varepsilon_{i,t}$$ (Eq 2)

Along with these variables, y_i,t_ for the explained variable, is the logarithm of farmers’ pure income. The key explanatory variable, δ_i,t_, is the poverty reduction policy. Similar to the setting of policy variables in the breakpoint regression, i.e. to set the value as 1 when the variable is no less than 2011 and set as 0 when it's less than 2011. D is the permanent influence of the poverty mitigation policy. $x_{i,t}^{\prime }$ is the control variable, particularly, the degree of market profitable activity and output value of agriculture in destitute location. Z_i,t_ is the instrumental variable of the explained variable δ_i,t_D. The first instrumental variable is the road network density of the poor country; the second is the second-order lag of farmers' per capita net pay (logarithm). μ_i_ pertains to the disturbance that signifies the diversity of the region. ε_i,t_ refers to the disturbance factor that varies depending on location and time.

We have used two instrumental variables: the first is the road network density of the poor counties; the second is the second-order lag of farmers' per capita net earnings. The reasons are as follows: Firstly, the road network density of poor districts is a distance unit, which is a purely exogenous variable. It is related to the poverty alleviation policy, because these counties, being located in the mountains with inconvenient transportation, were supported by the focused poverty mitigation policy. Secondly, the second-order lag of farmers' per capita net income had already occurred viewed as “pre-determined”. That means its value has been fixed from the current point of view without relationship to the poverty alleviation policy. Therefore, it is an exogenous variable. The second-order lag of farmers' individual net earnings related to the poverty alleviation because that of in impoverished counties implemented by the poverty alleviation policy is far lower than the national poverty line [33].

### Empirical results of the instrumental variable panel regression

In order to control the individual differences in geographical locations and climate of the impoverished districts in the TGRR, we have considered regional effects. The calculated outcomes are presented in Table 3.

**Table 3:** Instrumental variable panel regression results.

lnrural	Coef.	St.Err
dum	0.219957*	0.1084916
lngross	0.5740493*	0.2949775
lntotal	0.3682796**	0.1424943
_cons	-3.681508	1.9846171

Note

*, **, and *** represent the results are statistically significant at the levels of 5%, 1% and 0.1%, respectively.

Table 3 indicates that the poverty reduction policy (dum) is significantly at the 5% level, namely: 0.220. The policy impact of the China’s poverty reduction policy in 2011–2015 is 0.220. The market profitable activity (lntotal) is significantly at 1% level, whose value is 0.37. The agricultural GDP (lngross) is significantly at 5% level, whose coefficient is 0.57. These results indicate that greater success in market economy equates to more rapid increases in the rural individual net earnings. Furthermore, with better growth of total agricultural production in destitute areas, the faster the net income per capita is boosted. Weak instrumental variable tests are presented in the S1 Appendix and indicate that these two instrumental variables (road network density and second-order lagging net income per capita) pass the test, indicating that they are not weak instrumental variables.

### Comparative assessment of the focused poverty mitigation policy

The immediate and the lasting impacts of the specific poverty reduction policy were compared with the differences between the impoverished areas in the TGRR before and after the implementation of the policy, as above. However, to fully understand the effect of this policy, we have to compare its influence on areas where the policy was implemented and not. This enables us to comprehensively evaluate the contribution of the implementation of the policy to poverty reduction in the poverty-stricken areas. We conducted a comparative analysis between non-poor counties (control group) which had not implemented the targeted poverty alleviation policy and poverty-stricken counties (treatment group) where targeted reduction policy has been enforced. The comparative assessment can further verify the impacts of China’s targeted poverty mitigation policy.

Comparative analysis is better if the treatment group and the control group are as similar as possible except for the intervention being studied in the treatment group [34]. We regarded the 9 impoverished districts in the TGRR of Chongqing as the treatment group, and the remaining other counties (12 non-poverty counties) in the area as the control group. This is because their socio-economic conditions are very similar, apart from the enactment of the focused poverty reduction policy.

We adopted the generalized synthetic control method to conduct the comparative assessment of the focused poverty mitigation policy. It is a causal inference method, which is often used to assess the effects of policy implementation in a given area. This approach connects the two methods and combines the synthetic control method [35] in combination with the linear fixed effects models in an intricate framework [34]. Accordingly, our principle approach followed three key steps. Firstly, we averaged the 9 impoverished districts in the areas to construct an integrated treatment group. Secondly, we synthesized a control group by way of data-driven, and a linear combination of the 14 non-impoverished counties in the same region. The overall socio-economic conditions of the control group were the same as the treatment group before the enforcement of the focused poverty reduction policy (1998–2010), so the control group played the counterfactual agent for the treatment group. Throughout the execution of the focused poverty mitigation policy (2011–2015), we assessed its impacts by comparing the differences between the impoverished districts in the TGRR and the non-poverty counties in this region. Thirdly, the generalized synthetic control method involves a cross-validation scheme integrating the IFE (Interactive Fixed Effects) model and the synthetic control method. It can determine the influence of control variables on the dependent variable. Therefore, we added the control variables, i.e., market profitable activities and total agricultural production in order to verify whether market economic activities, total agricultural production and road network density in the destitute regions still affected the individual net earnings of farmers, compared with those in non-poor counties in the TGRR. The empirical results of the comparative assessment of the policy are exhibited in Table 4, while that of the poverty reduction effects of controlled variables are displayed in Table 5.

**Table 4:** Empirical results of comparative assessment.

	ATT	S.E.	n.Treated
Total	0.07029***	0.024760	1
1998	0.0175472	0.005252	0
1999	-0.0061936	0.003853	0
2000	-0.0101421	0.003216	0
2001	-0.0074791	0.003542	0
2002	-0.0027808	0.002584	0
2003	0.0024271	0.003274	0
2004	0.0045210	0.003434	0
2005	-0.0016047	0.004340	0
2006	0.0001307	0.003721	0
2007	-0.0021429	0.002888	0
2008	-0.0004874	0.003089	0
2009	0.0011818	0.003718	0
2010	0.0053503	0.007506	0
2011	0.0517023*	0.025793	11
2012	0.0620097*	0.027296	11
2013	0.0697447**	0.028524	11
2014	0.0759275**	0.030670	11
2015	0.0920832***	0.030831	11

**Note:** (1) ATT (Average Treatment Effect on the Treated) is the Mean Treatment Effect for the Treatment Group; S. E. is a standard error; n Treated = 0 represents the poverty reduction policy has not yet been enacted. n Treated = 1 represents the enforcement period of this policy. Total represents the total treatment effect for 2011–2015 during the implementation e policy. 2011–2015 represent the annual treatment effect of 2011–2015, respectively. (2) *, **, and *** indicate the results are statistically significant at the levels of 5%, 1% and 0.1%, respectively.

**Table 5:** Poverty reduction effect of covariate variables.

	beta	S.E.
lntotal	0.06028426**	0.04463370
lngross	0.07290472***	0.03268858
densityroad	0.03536731*	0.02016181

**Note:** (1) *, **, and *** indicate the results are statistically significant at the levels of 5%, 1% and 0.1%, respectively.

Table 4 indicates that through the comparative analysis of poverty-stricken and affluent districts in the TGRR, the implementation period of the policy (2011–2015) still has a substantial influence on poverty mitigation, wherein the poverty reduction effect of the policy is 0.07. It means that the increase of farmers’ net income in the poverty-stricken counties is 7% higher than that of non-poverty counties. Specifically, compared with those of the non-poor counties, the poverty mitigation effects of the impoverished counties in the TGRR are 5.2%, 6.2%, 7%, 7.6% and 9.2% higher for the years in 2012, 2013, 2014, and 2015 respectively.

Table 5 indicates that compared with those in the rich districts in the TGRR, the poverty reduction effects of market economy activity (lntotal), total agricultural output value (lngross), and road density (densityroad) in the poor counties of the region are still significant. The poverty reduction effect of the market economy activity is 0.06, that of the total agricultural output value is 0.07, and that of the road network density is 0.04. They are significant at the levels of 1%, 0.1%, and 5%, respectively. This result shows that, as compared with the development of non-poor counties, the more active of the market economy in the poor counties, the faster the rise of the individual net earnings of farmers. Likewise, the higher the road network density in the destitute locations, the faster the increase of the farmers’ net pays.

The development in the level of market activities in the poverty-stricken region helps to reduce poverty. The reason is that the increase in consumer products’ overall retail sales in the poor areas will result in an increase in the volume of commodities traded in this area. It also means that more agricultural products were involved in market transactions, indicating that individual farmers, including the rural poor, have a better access to the market, which is conducive to improving farmers' income especially cash income from farm products for the poor.

## Discussion

We have used the causal inference research methods-breakpoint regression and instrumental variable regression to dynamically analyze the short-term and permanent effects of China's focused poverty mitigation policy. And then we have adopted a comparative study to further verify the poverty reduction effect of the policy. Compared with other scholars in the past, the uniqueness of our study is obvious. Firstly, when studying the short-term effects of the policy, we abandoned the subjective research method i.e. establishing an indicator system to study the effectiveness of poverty alleviation policies. We adopted an objective method—breakpoint regression to objectively obtain the short-term causal effect of the policy. And then we used a data-driven approach to study the time of the poverty reduction effect in the initial stage. Secondly, when studying the long-term effects of the policy, we used the causal inferred research method—instrumental variables regression to obtain the long-term causal effects of the policy. Thirdly, we studied the immediate and durable effects of the poverty reduction policy by comparison of the changes of poverty-stricken counties before and after the implementation of the policy, and integrated these effects into the poverty alleviation policy research framework. Fourthly, through the comparative research of poor and non-poor counties, we adopted the generalized comprehensive control method for the first time which is a causal inference frontier method, to further verify the poverty mitigation effect of the poverty reduction policy.

The significance of this study is to provide a systematic analysis framework and policy recommendations for the assessment of poverty reduction policies. That is: firstly, we have integrated the temporary and lasting impacts of the poverty reduction policy and verified the poverty reduction effect, providing a policy evaluation framework. Secondly, we have innovatively integrated causal inference research methods such as instrumental variable panel regression, breakpoint regression and generalized comprehensive control method to construct the policy effect assessment framework from the long-term and short-term effects, in an attempt to assess the actual impacts of China’s poverty mitigation policies.

## Conclusions and recommendations

### Conclusions

(1) China’s policy for focused poverty reduction has substantial poverty mitigation effects.

The extent of the focused poverty mitigation policy was from 2011 to 2015, wherein the policy impacts was observed at the initial phase and persisted as its implementation continued. The temporary and permanent policy impacts were obtained from breakpoint and instrumental variables panel regression analyses. Results indicate that the impacts at the start (i.e., from 2011 to 2013) and throughout the five-year (2011–2015) policy enforcement were 0.139 and 0.220, accordingly. During the initial stages of policy enforcement from 2011 to 2013, the net earnings of each farmer in the district rose by 13.9% compared with 2008–2010. After its implementation, from 2011 to 2015, the individual net earnings of farmers improved by 22% in contrast to the period from 1998 to 2010 when the focused poverty mitigation policy was not enacted.

(2) The comparative studies find that China’s focused poverty reduction policy has a significant poverty mitigation impact.

The comparative studies between poor and non-poor counties show that the implementation period of the policy (2011–2015) still has a substantial influence on poverty alleviation, wherein the poverty reduction effect of the policy is 0.07. That means that compared with the non-poor counties in the TGRR, the enforcement of the focused poverty reduction policy has increased the individual net earnings of farmers by 7%. The study further shows that this policy has a considerable poverty reduction effect.

(3) Market profitable activity as well as agricultural GDP have an effect on the farmers’ individual net earnings in the poor districts.

The outcomes of instrumental variables panel regression suggest that in terms of the long-term development of poverty alleviation, additional 1% in market profitable activation and gross agricultural production makes it available that poverty stricken population can gain increase in net earnings of every farmer by 0.37% and 0.57% respectively. Therefore, for sustainable poverty alleviation, market invigoration and farming augmentation are necessary.

### Policy recommendations

(1) For poverty relief in the most poverty-stricken areas, China should increase investment to consolidate its poverty alleviation policy. Our empirical findings depict that China’s poverty mitigation policy has a substantial impact on improving the individual net earnings of farmers in destitute districts. Because of the destitute natural environment, weak economic foundation and deep degree poverty in the most poverty-stricken areas, it is very difficult to rely on self-development to shake off poverty. China should increase capital investment in the most poverty-stricken areas, increase new poverty-reduction funds, increase the scale of transfer payments in order to form a powerful force to accelerate poverty eradication of poverty-stricken areas especially in the Three Gorges Reservoir Region.

(2) China should vigorously develop the market in poor areas and reduce poverty through market development.

The measures of vigorously develop markets in impoverished areas include that (i) public expenditures and investments from state-owned economic entities maintain the overall social investment ratio to drive continuous growth in total demand; (ii) various economic means strengthen macroeconomic regulation, curb deflation and inflation, and avoid substantial economic growth fluctuation; (iii) gradually improvement of the market system, and stimulation of the technological innovation of market players in poor areas to improve production efficiency.

(3) As part of the government effort, agricultural production should be developed at local level to accelerate overall poverty alleviation in poor-stricken areas. Our research results indicate that agricultural production in poor areas can increase the net earnings of each farmer. Therefore, it is necessary to develop agriculture in accordance with local production conditions and geographical characteristics, promote the development of high-efficiency agriculture, ecological agriculture, characteristic agriculture and modern agriculture, and build characteristic agriculture and rural economy in order to accelerate the way out of poverty.

## Supporting information

S1 Appendix (DOCX)
