# Supplementary material for: Probe into the targeted poverty mitigation policy in China based on causal inference: Evidence from Chongqing in the Three Gorges Reservoir region
Source: PLoS One. 2021 Jan 5;16(1):e0244928. doi: 10.1371/journal.pone.0244928 (PMC7785243; doi:10.1371/journal.pone.0244928)
Supplement: S1 Appendix — (DOCX) [file pone.0244928.s001.docx]

S1 Appendix for

Probe into Focused Poverty Mitigation Policy in China on the Basis of Causal Inference: Proof of Chongqing in the Three Gorges Reservoir Region

**Table of Contents**

[Robustness test of the findings from the breakpoint regression 1](#_Toc40605563)

[Weak Instrumental Variable Tests 2](#_Toc40605564)

Robustness test of the outcomes of the breakpoint regression

There is the hypothesis for casualty estimation via regression discontinuity is that the jump of the variable varies with the driving variable and is independent of the covariant, thus, we took all covariants as outcome variables for the regression. As shown in table A1, all covariants do not show a significant change before and after the policy implementation of the focused poverty mitigation, which also proves that the estimation is effective.

**Table A1.** Robustness Tests Results for the Breakpoint Regression

|  | **(1)**  **lngross**  **(tri)** | **(2)**  **lngross**  **(epa)** | **(3)**  **lntotal**  **(tri)** | **(4)**  **lntotal**  **(epa)** |
| --- | --- | --- | --- | --- |
| Conventional | -0.119  (0.371) | -0.119  (0.370) | -0.025  (0.528) | -0.024  (0.527) |
| Bias-corrected | 0.029  (0.371) | 0.029  (0.370) | 0.094  (0.528) | 0.094  (0.527) |
| Robust | 0.029  (0.257) | 0.029  (0.256) | 0.094  (0.372) | 0.094  (0.372) |

**Note:** (1) *, **, and *** indicate the results are statistically significant at the levels of 5%, 1% and 0.1%, respectively. (2) tri is triangular kernel; epa is quadratic kernel. Lngross represents total amount of agricultural produce in impoverished districts; lntotal represents the market economic activity.)

Weak Instrumental Variable Tests

If the instrumental variable is a "weak instrumental variable", the asymptotic variance of the estimator from the instrumental variable method will become very large and the estimate will be biased [1]. Therefore, the test for weak instrumental variable is required. The empirical outcomes are presented in Table A2. It depicts that the value of Robust F is 13.127 greater than 10. This means that two instrumental variables (road network density and second-order lagging net income per capita) are not weak instrumental variables. Therefore, it has passed the test of week instrumental variable.

**Table A2.** Weak Instrumental Variable Tests

| Variable | R-sq | Robust F(2,49) |
| --- | --- | --- |
| dum | 0.8151 | 13.1272 |

References

[1] Angrist, J.D., Pischke, J.S. Mostly harmless econometrics: An empiricist's companion. New Jersey: Princeton university press**.** 2008
